# The AII amacrine cell connectome: a dense network hub

**DOI:** 10.3389/fncir.2014.00104

**Published:** 2014-09-04

**Authors:** Robert E. Marc, James R. Anderson, Bryan W. Jones, Crystal L. Sigulinsky, James S. Lauritzen

**Affiliations:** Department of Ophthalmology and Visual Sciences, John A. Moran Eye Center, University of Utah School of MedicineSalt Lake City, UT, USA

**Keywords:** connectomics, retina, networks, hub, bipolar cells, amacrine cells, synapses, gap junctions

## Abstract

The mammalian AII retinal amacrine cell is a narrow-field, multistratified glycinergic neuron best known for its role in collecting scotopic signals from rod bipolar cells and distributing them to ON and OFF cone pathways in a crossover network via a combination of inhibitory synapses and heterocellular AII::ON cone bipolar cell gap junctions. Long considered a simple cell, a full connectomics analysis shows that AII cells possess the most complex interaction repertoire of any known vertebrate neuron, contacting at least 28 different cell classes, including every class of retinal bipolar cell. Beyond its basic role in distributing rod signals to cone pathways, the AII cell may also mediate narrow-field feedback and feedforward inhibition for the photopic OFF channel, photopic ON-OFF inhibitory crossover signaling, and serves as a nexus for a collection of inhibitory networks arising from cone pathways that likely negotiate fast switching between cone and rod vision. Further analysis of the complete synaptic counts for five AII cells shows that (1) synaptic sampling is normalized for anatomic target encounter rates; (2) qualitative targeting is specific and apparently errorless; and (3) that AII cells strongly differentiate partner cohorts by synaptic and/or coupling weights. The AII network is a dense hub connecting all primary retinal excitatory channels via precisely weighted drive and specific polarities. Homologs of AII amacrine cells have yet to be identified in non-mammalians, but we propose that such homologs should be narrow-field glycinergic amacrine cells driving photopic ON-OFF crossover via heterocellular coupling with ON cone bipolar cells and glycinergic synapses on OFF cone bipolar cells. The specific evolutionary event creating the mammalian AII scotopic-photopic hub would then simply be the emergence of large numbers of pure rod bipolar cells.

## Introduction

**Figure 1:**
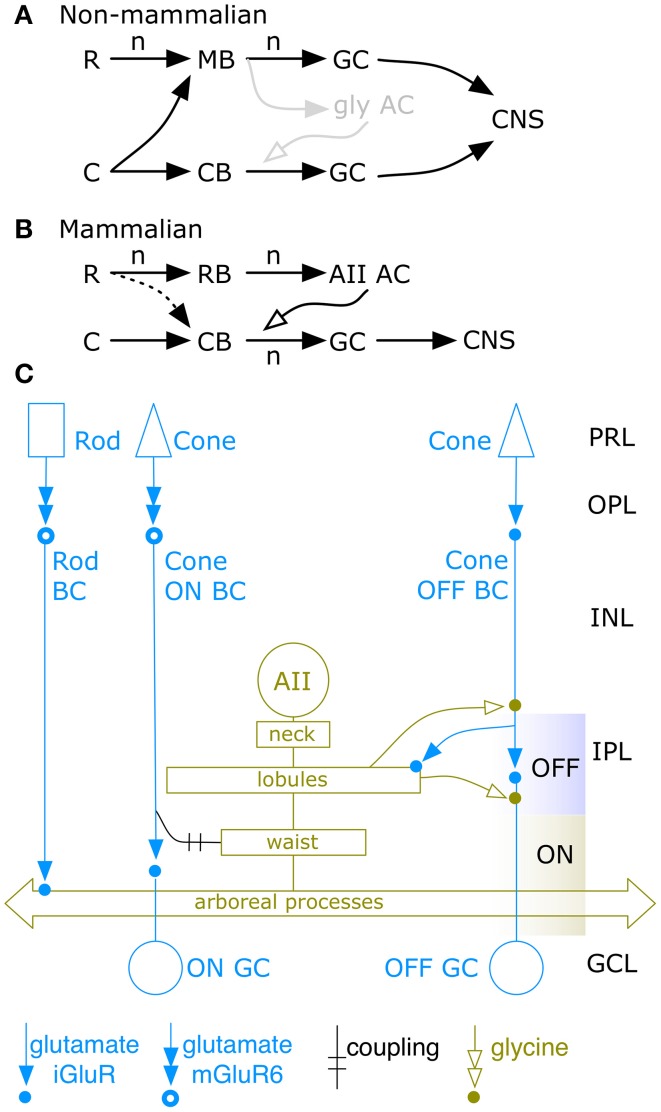
**Basic AII cell networks**. Vertebrate rod R and cone C signal convergence patterns onto bipolar cells, amacrine or ganglion cells, and CNS targets. **(A)** Non-mammalian networks display two channel types: pure cone bipolar cell (CB) and mixed rod-cone bipolar cell (MB) channels that drive sets of retinal ganglion cells (GC) projecting to CNS targets via high gain (n) glutamate signaling (black arrows). The non-mammalian rod to GC chain has a net gain of *n*^2^. **(B)** Mammalian networks display separate CB and rod bipolar cell (RB) channels. Only CB channels drive GCs. RB channels drive only amacrine cells (ACs), in particular the AII AC that provides low gain coupling or glycinergic signaling (open arrow) from the third stage back to stage two in the CB chain. The mammalian rod to GC chain has a net gain of *n*^3^. There is evidence for sparse rod signal leakage into the CB chain. The gray glycinergic (gly) motif in **(A)** is the hypothetical evolutionary precursor of the mammalian AII AC. **(C)** The classic AII amacrine cell network, *circa* 1992. The rod input is collected by rod bipolar cells (Rod BC) which drive AII cells by ionotropic glutamate receptors (iGluRs). Cone input is collected by OFF cone bipolar cells (OFF BC) that also sparsely drive AII cells by iGluRs. The AII network is extensively coupled to ON cone bipolar cells. Glycinergic output from the AII network targets OFF BCs and OFF ganglion cells (OFF GC).

The network spanning photoreceptor input and ganglion cell output in the mammalian retina was detailed by Kolb and Famiglietti (1974) using serial section transmission electron microscope (TEM) imaging. Unlike non-mammalian retinal networks (Famiglietti et al., 1975; Naka et al., 1975), mammalian photoreceptor networks were parsed into discrete cone and rod bipolar cell pathways and, remarkably, rod-driven bipolar cells did not synapse on ganglion cells. So how would scotopic signals reach the brain? The solution was a unique interneuron, the AII amacrine cell (Figures 1A,B), which captured rod bipolar cell input and redistributed it to cone bipolar cells (Figure 1C), using the synaptic endings of cone bipolar cells as adaptors. This motif was unprecedented in any CNS network: a stage in an afferent amplification chain acting as the entire signal output for a qualitative channel to a prior parallel stage, effecting divergence of one signal into channels primarily used by another signal, with additional amplification. While reentrant CNS motifs are well-known, e.g., layer 6 corticothalamic projections (Da Costa and Martin, 2009), the outflow pattern of the AII cell is unique in its scope and nature. Reconstruction and tabulation of synaptic flow in a single AII amacrine cell was achieved by Strettoi et al. (1992). These TEM studies of AII amacrine cells described an architecture and synaptic partnerships that still cannot be explained by or predicted from physiological data. Conversely, while some features of AII cell connectivity broadly predict its physiological responses, no complete model emerges from these anatomical data. On balance and despite its extensive analysis, the evolution, functional scope and connectivity of the AII amacrine cell remains unclear.

Why do we care about the AII cell at all if alternative paths bypass rod bipolar cells? Put simply, AII paths dominate scotopic vision and appear to set the scotopic threshold. Alternative paths access cone bipolar cells via presumably weaker paths, e.g., small gap junctions between rods and cones (Massey, 2008) or sparse direct contacts with OFF cone bipolar cells (Devries and Baylor, 1995; Soucy et al., 1998; Tsukamoto et al., 2001; Pang et al., 2010, 2012) or ON cone bipolar cells (Tsukamoto et al., 2007; Pang et al., 2010). AII and rod bipolar cells comprise a great fraction of their cognate groups and vastly outnumber those OFF bipolar cells thought to receive rod input (Pang et al., 2012). The AII network has a unique mechanism for achieving the high sensitivity characteristic of mammalian scotopic vision (e.g., Saszik et al., 2002; Frishman, 2006). Finally, threshold scotopic OFF responses of retinal ganglion cells are blocked by strychnine, implying a dominant glycinergic drive, consistent with the key role of AII cells in the network (Muller et al., 1988; Arman and Sampath, 2012).

The rod::cone coupling pathway is nominally shared across vertebrates (e.g., Attwell et al., 1984) but there is no evidence that it accounts for the high scotopic sensitivities of mammals. Further, rod convergence onto bipolar cells in mammals is not homologous to the mixed rod-cone bipolar cell cohorts of non-mammalians. The mammalian retina is rod dominated but rod contacts with OFF bipolar cells (Figure 1B) are sparse (Tsukamoto et al., 2001) and can even be missing within target OFF bipolar cell classes (Li et al., 2010). Rod input to OFF cone bipolar cells in mammals also appears restricted to a one class of bipolar cell in mouse (Pang et al., 2012) and appears constrained to flow to only a subset of target ganglion cells (Devries and Baylor, 1995; Wang, 2006). In contrast, ectotherms exhibit multiple classes of rod-dominated bipolar cells (Figure 1A) that have precise amounts of cone input (Scholes and Morris, 1973; Scholes, 1975; Ishida et al., 1980). Further, the mixed rod-cone ON pathway in teleost fishes uses different transduction mechanisms for rods and cones (Grant and Dowling, 1996) with distinct positive cationic and negative anionic reversal potentials for rods and cones respectively (Saito et al., 1979). No such weighting or specific transduction appears in mammals. Thus, is it unlikely that the alternative mammalian pathways approach the sensitivity of the AII system. Mammals show high scotopic sensitivity and the sensitive STR (scotopic threshold response) waves of the mammalian electroretinogram are APB-sensitive and kinetically slow, implying the STR depends on rod bipolar cells and, likely, AII cells (Saszik et al., 2002; Frishman, 2006). The unique AII cell and its connectivity thus remains of central interest in the evolution of mammalian rod vision.

Our approach to this problem is based on automated TEM (ATEM) connectomics. ATEM connectomics enables the acquisition of rich synaptic maps by characterizing all partners and structural weights for all synapses and synapse types using connectome volume RC1. Connectome volume RC1 is a synaptic resolution ATEM dataset from rabbit retina spanning the inner nuclear, inner plexiform and ganglion cell layers of a sample field 0.243 mm in diameter. It currently contains ≈890,000 annotations; ≈600 identified neurons; 6500 conventional and 13,700 ribbon synapses; 26,000 identified postsynaptic sites, over 3800 gap junction pairs, and 2280 adherens junctions; all assembled into 8600 identified presynaptic/postsynaptic partnerships. Volume RC1 specifically contains 39 verified AII amacrine cells, 104 rod bipolar cells, ≈300 cone bipolar cells, and ≈200 amacrine cells, with processes from many more amacrine cells entering the margins of the volume (Anderson et al., 2011b; Marc et al., 2013). This provides us with the opportunity to characterize the complete AII amacrine cell morphology, synaptology, network motifs and synaptic weighting. We have reconstructed 5 cells to statistical completion with large portions of all 39 mapped. This has permitted definitions of all AII partner classes and establishes key synaptic weights for a more comprehensive model of AII cells.

## Materials and methods

The methods for connectome RC1 have been extensively detailed by Anderson et al. (2009, 2011a,b). The RC1 dataset is freely available at connectomics.utah.edu. The associated software is available as free (SerialEM) or open-source applications (Nornir build manager, nornir.github.io/nornir-buildmanager), or via a free license (Viking and Viz web-services tools) for educational use through the University of Utah. The raw RC1 dataset is available on user-provided storage media.

### Tissue harvest and processing

The retinal sample for ATEM image volume RC1 was acquired from a euthanized light-adapted female Dutch Belted rabbit (Oregon Rabbitry, OR). All protocols were in accord with Institutional Animal Care and Use protocols of the University of Utah, the ARVO Statement for the Use of Animals in Ophthalmic and Visual Research, and the Policies on the Use of Animals and Humans in Neuroscience Research of the Society for Neuroscience. At euthanasia, the eye was injected with 0.1 ml fixative with 18 gauge needle pressure relief, enucleated, hemisected, and fixed 24 h in 1% formaldehyde, 2.5% glutaraldehyde, 3% sucrose, 1 mM MgSO_4_, in 0.1 M cacodylate buffer, pH 7.4. Dissected, isolated retinal pieces were immersed in 0.5% OsO4 in 0.1 M cacodylate buffer for 60 min, processed in maleate buffer for *en bloc* staining with uranyl acetate, and processed for resin embedding (Marc and Liu, 2000; Anderson et al., 2009). Retinal blocs were serially sectioned in the horizontal plane at 70–90 nm on a Leica UC6 ultramicrotome onto carbon-coated Formvar® films supported by gold slot grids. Optical 70–90 nm sections were captured and processed for computational molecular phenotyping (CMP) as defined previously (Marc et al., 1995; Marc and Jones, 2002) by probing with anti-hapten IgGs targeting small molecules: GABA, glycine, glutamate, glutamine, or taurine (Signature Immunologics Inc, Salt Lake City, UT). Small molecule signals were visualized with silver-intensification of 1.4 nm gold granule-conjugated goat anti-rabbit IgGs (Nanoprobes, Yaphank, NY). Optical (8-bit 1388 pixel × 1036 line frames) images were captured, mosaicked, aligned, and processed for classification (e.g., Marc and Jones, 2002; Anderson et al., 2009). Volume RC1 was bracketed by 10-section optical CMP series and intercalated every 30 sections with one CMP section. This inserted definitive molecular signals into every retinal neuron. The final dataset spanned 401 sections.

### Volume assembly

RC1 was created as previously described (Anderson et al., 2009, 2011b; Lauritzen et al., 2012). Briefly, the desired field on each grid was captured by SerialEM (Mastronarde, 2005; Anderson et al., 2009) using a Gatan US4000 phosphorimaging camera. Each capture field is an array of ≈1000 tiles captured at 2.18 nm resolution. Mosaics and 3D volumes were originally generated using the NCR Toolset (http://www.sci.utah.edu/download/ncrtoolset). This code has now been superseded by Nornir (nornir.github.io/nornir-buildmanager/). CMP-to-TEM registrations are operator-guided with ir-tweak software from the NCR toolset. Re-imaging for optimized resolution and section tilt is performed using using high resolution (20,000–60,000×) goniometric tilt series.

### Image viewing, annotation, and analysis

Volume RC1 was visualized and annotated with the Viking viewer (Anderson et al., 2011a). The annotations trace 3D cell architectures as well as locations and dimensions of presynaptic, postsynaptic, adherens, and gap junction motifs as well as non-junctional touches are logged in the Viking database and visualized using VikingPlot, a compiled Matlab application that queries structure information from the annotation database and renders surfaces for display. VikingPlot exports formats for rendering of 3D data in a variety of free (e.g., Blender, blender.org) and commercial applications. The annotation database permits standard SQL queries.

### Image preparation

Publication figure preparation followed Anderson et al. (2009, 2011b). Raw optical image data are available upon request and RC1 is public-access. Multi-modal registered optical images were max-min contrast stretched and sharpened using unsharp masking at a kernel extent of ≈540 nm. While ATEM images after NCRToolset and Nornir processing tend to have high contrast, none of the Viking ATEM data shown here except for **Figures 9B–D** have been processed. Overlay methods for combining optical and TEM images generally computed HSB values for a new image using the TEM gray scale brightness (B) and hue and saturation from (H,S) from the rgb optical image. Occasionally, fourth or fifth channels were added using alpha blending. Renderings of structures in VikingPlot were created in Matlab 2009a as described in Anderson et al. (2011b).

### Dataset analysis

Every cell annotation in RC1 is a 2D disc that is the largest inscribed circle contained by the close shape of the cell's margins (typically a star domain) in a given slice. Internal structures such as gap junctions, presynaptic specializations, postsynaptic densities (PSDs), etc., are linked to a cell via child annotations: 2D discs representing the child structure's Feret diameter. These annotations, summed over slices, enable quantitative assessments of features (e.g., gap junction and PSD areas) and 3D representations of cells and child structures. These data are accessed via Viking Viz (Anderson et al., 2011a) or Microsoft SQL queries. Large queries were exported as delimited text files. We used AnalystSoft, StatPlus:mac (www.analystsoft.com) for statistics and histogramming.

## Results

### Basic attributes of AII cells

**Figure 2:**
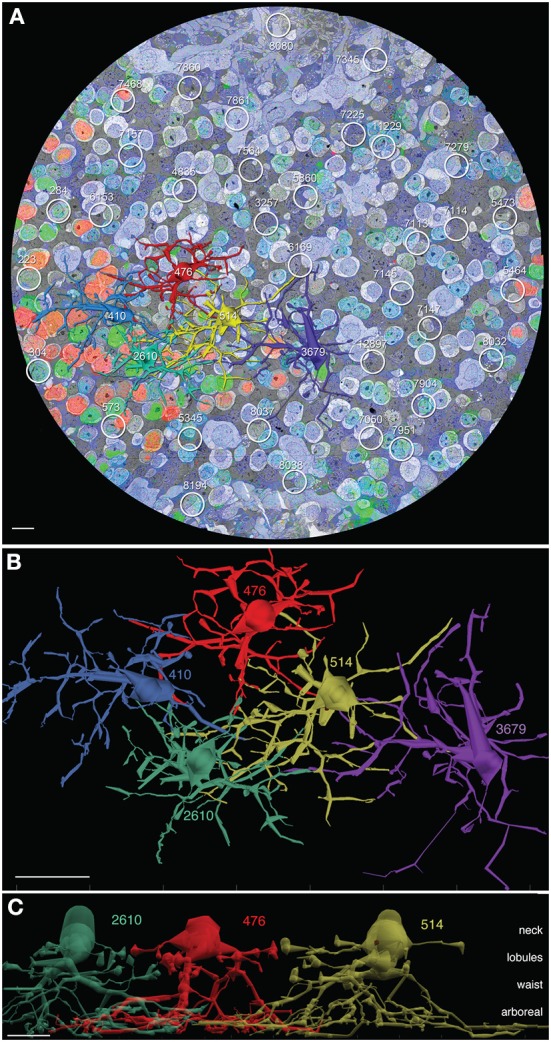
**AII cells in connectome RC1. (A)** Slice z001 with 5 channel molecular overlay (see Anderson et al., 2011b), 5 rendered AII cells (410 blue, 476 red, 514 gold, 2610 green, 3679 purple) and 34 other AII loci indicated by circles. **(B)** Top view (XY plane) of VikingPlot rendered AII amacrine cells. **(C)** Side view of AII amacrine cells 2610, 476, and 514 laterally displaced to reveal the neck, lobule, waist, and arboreal zones. Scales **(A,C)** 10 μm; **(B)** 20 μm.

**Figure 3:**
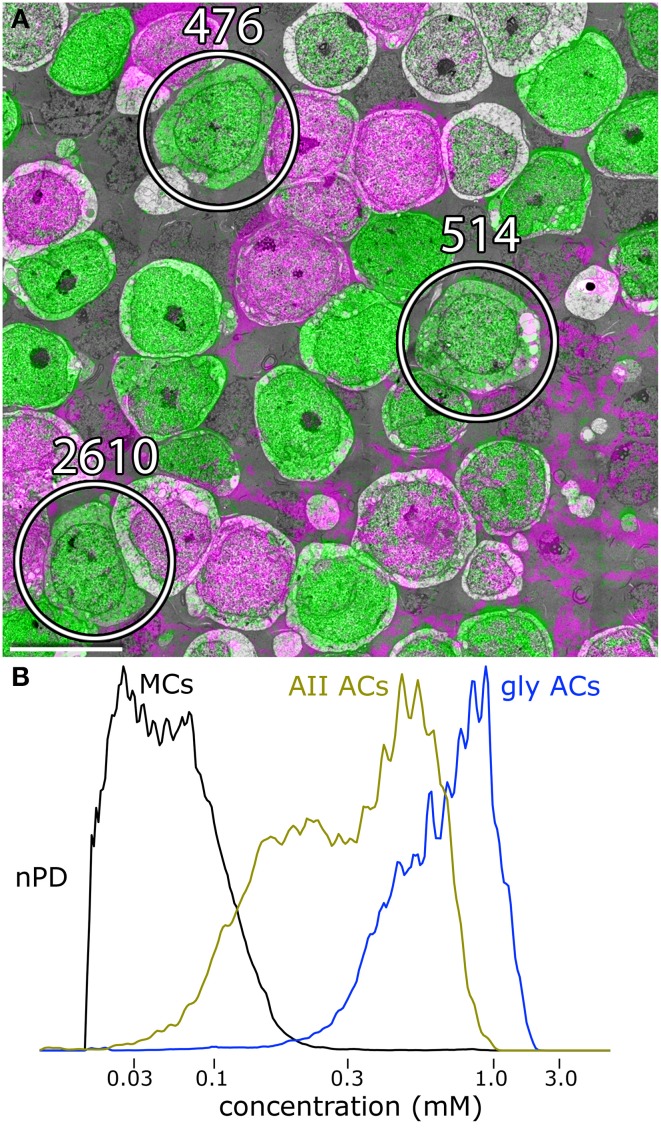
**Glycine and GABA signals in amacrine cells. (A)** Slice 030, volume RC1. Three of the AII amacrine cells are marked. The gray space in between cells is filled with glial Müller cell processes. The pale gray cells are bipolar cells. Scale 10 μm. **(B)** Glycine content histograms distinguish AII cells from other retinal cells. The glycine content of AII cells (AII AC, gold trace) is about 2-fold lower than all other glycinergic amacrine cells combined (gly ACs, blue trace), but over 20-fold higher than signals in glial Müller cell (MCs). The histograms each represent aggregate signals from 25 cells calibrated as described in Marc and Jones (2002) and displayed as normalized probability density (nPD) vs. pixel value scaled as concentration.

The analysis of AII cells in RC1 includes (1) mapping all AII somas and domains, (2) complete 3D reconstruction of five specific AII neighboring cells (cells 410, 476, 514, 2610, and 3679), (3) classification of contacts with AII cells, and (4) mapping all synapses and gap junctions made by discrete AII cells. Volume RC1 contains 39 AII cells (Figure 2A) corresponding to a density of 841 cells/mm^2^ with a center-to-center tile spacing of 34 μm. The nearest-neighbor soma-to-soma spacing is 30 ± 8 μm (mean ± 1 standard deviation, *n* = 39 pairs), consistent with the fact that somas are rarely positioned over the center of a Voronoi tile (e.g., Figure 3B) and the average jitter is about 10%. Five AII cells were selected for detailed analysis (Figure 2B).

AII cells in RC1 have distinctive features that enable unambiguous classification. They are narrow field glycinergic amacrine cells with somas of ≈8–10 μm in breadth and thick necks ≈5–8 μm in diameter that extend deeply into the inner plexiform layer (Figure 3C). The neck is a target of four to six large synapses from TH1 dopamine/glutamate neurons (Anderson et al., 2011b) and also extends five or six thin stalks that extend about 10–20 μm laterally and form irregular synaptic vesicle-rich lobules, often prolate in shape with a major axis of ≈3 μm and a minor axis ≈2 μm. At the base of the neck, four or five thick arboreal dendrites emerge and can branch once or twice, forming a conical waist about 30 μm in diameter as they descend obliquely the inner plexiform layer to the proximal margin where rod bipolar cells provide direct synaptic input over a field ≈60 μm wide. Each of these zones, the neck, lobules, waist, and arboreal terminal branches, have distinctive connectivities that are zone-specific, not merely encounter-specific. We will discuss this more extensively below. But, as an example, rod bipolar cell axons touch AII lobules in passage yet never form the connections that are found between AII arboreal processes and rod axons or axon terminals.

**Figure 4:**
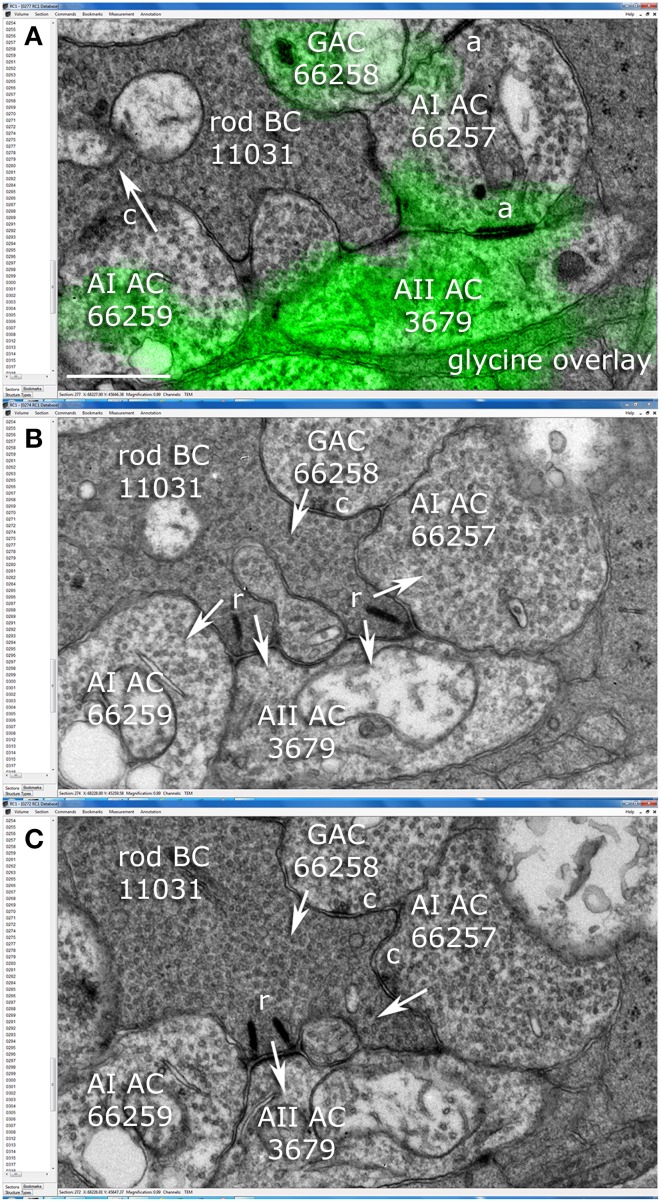
**Synaptic relationships among amacrine and rod bipolar cells in the Viking connectome viewer. (A)** Slice z277: overlay of glycine channel and TEM. AI cell 66257 forms adherens junctions (a) with AII cell 3679 and glycinergic amacrine cell (GAC) 66258. AI cell 66259 forms a conventional synapse (c) onto postsynaptic target (arrow) rod bipolar cell (rod BC) 11031. **(B)** Slice z274: Rod BC forms two synaptic ribbons (r) onto postsynaptic targets AII 3679, AI 66257 and AI 66259.GAC 66258 is presynaptic to rod BC 11031. **(C)** Slice z272: rod BC forms a third ribbon with AII 3679 and AI 66257 forms a feedback synapse. Scale **(A)**, 500 nm.

AII cells are glycinergic, maintaining ≈0.6 mM cytoplasmic glycine. Volume RC1 is supported by capstone and intercalated ultrathin optical sections with an array of molecular markers. Figure 3A shows a small section of slice 030 of the inner nuclear layer displaying glycine (green) and GABA signals (magenta) in adjacent amacrine cells. Every AII cell was validated for glycine content by population histograms (Figure 3B). High contrast optical maps of small molecule signals are compliant with ATEM connectomics and significantly assist in tracking neural features (Marc and Liu, 2000; Jones et al., 2003; Anderson et al., 2011b; Lauritzen et al., 2012). Annotation of individual processes often intersects one of the intercalated molecular channels, enabling confirmation of identity, even at the limits of optical resolution. Figure 4 is a set of direct Viking images of serial sections through an AII cell arboreal dendrite as at traverses the surface of a rod bipolar cell. In slice z277 (Figure 4A), a strongly glycine positive arboreal dendrite of AII amacrine cell 3679 approaches one of its target rod bipolar cells (rod BC 11031), as well as a cluster of other amacrine cells. Notably, every AII cell makes large adherens junctions with AI amacrine cells (e.g., AI AC 66257), despite their structural and molecular diversities. As AII 3679 passes rod bipolar cell 11031, it makes three ribbon synapses in a span of 200–250 nm (z274 Figure 4B, z272 Figure 4C). This ribbon cluster also targets two AI cells, one of which is presynaptic to the rod bipolar cell in a classic reciprocal feedback motif. Nearby, a cone-driven glycinergic amacrine cell GAC 66258, part a major cone → rod crossover motif, is presynaptic to the rod bipolar cell.

**Figure 5:**
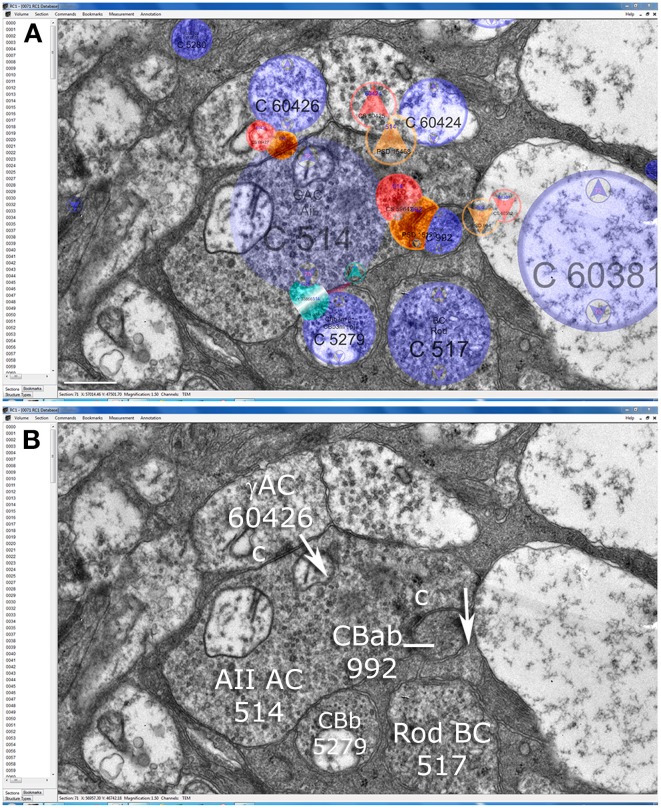
**Synaptic patterns of AII cell lobules in the Viking connectome viewer. (A)** Slice z71 showing annotation overlays for cells (blue), presynaptic elements (red), postsynaptic elements (orange) and touches (cyan). **(B)** A lobule from AII cell 514 is presynaptic to OFF cone bipolar cell CBab 992, postsynaptic to GABAergic amacrine cell (γAC) 60426, and touches ON cone bipolar cell CBb 5279 without making any other specialization. Scale **(A)**, 500 nm.

**Figure 6:**
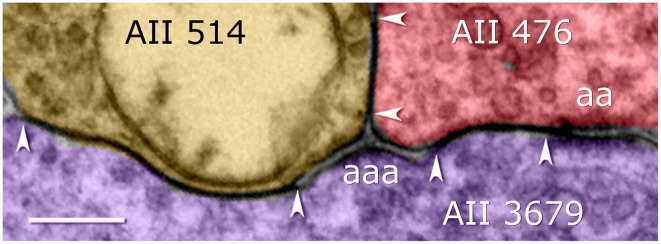
**Coupling among arboreal processes of AII cells in the Viking connectome viewer**. Contiguous gap junctions between arrowheads range 200–500 nm in extent. All gap junctions are accompanied by dual (aa) or triple (aaa) adherens junctions. Scale, 200 nm.

AII cells display their only synaptic output at the level of the synaptic lobules (Figure 5), which maintain vesicle densities as high as retinal bipolar cells (1488 ± 171 vesicles/um^3^, mean ± 1 standard deviation, *n* = 7 lobule sections excluding organelle volumes). The dominant targets of lobules are OFF cone bipolar cells, although AII cells also target specific OFF driven amacrine and ganglion cells (summarized below). AII lobules are complete integration sites as they are postsynaptic to OFF cone bipolar cells and several classes of GABAergic and glycinergic amacrine cells (Anderson et al., 2011b). However, as shown below, the amacrine cell input dominates by far and a typical AII cell can have has few as 3 OFF bipolar cell inputs or as many as 10. Finally, a distinctive feature of AII cells is their extensive homocellular and heterocellular coupling through large gap junctions made by their arboreal processes. Visualization of gap junctions requires at least 2 nm resolution. Figure 6 displays a triple gap junction complex formed by three of the mapped AII cells in this study. Coupling sites are always accompanied by distinctive adherens complexes. Heterocellular gap junctions are made between AII cells and a range of ON cone bipolar cells (Kolb and Famiglietti, 1974; McGuire et al., 1984; Strettoi et al., 1992; Anderson et al., 2011b; Marc et al., 2013).

### Large scale partnership mapping

**Figure 7:**
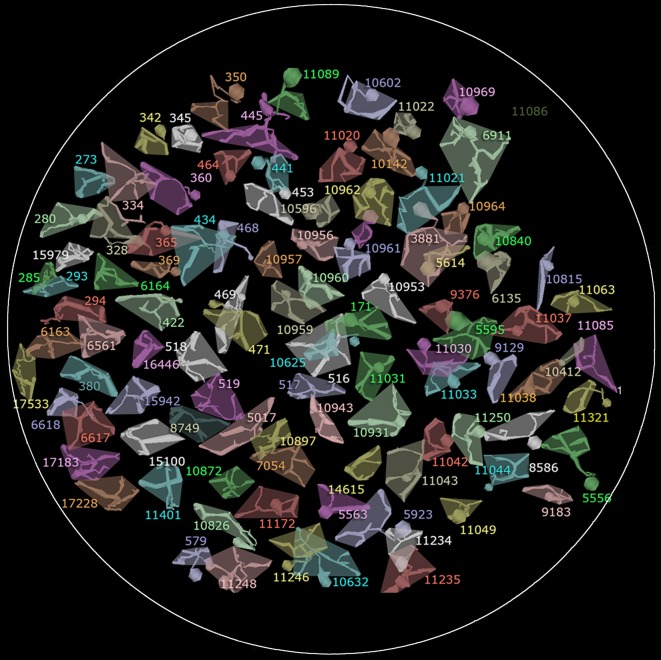
**The array of all 104 rod bipolar cell axonal fields in RC1**. The shaded polygon represents the convex hull for each cell. In some cases the soma and axon extend at an angle away from the axonal field and are not included in the hull. Circle scale, 0.25 mm.

**Figure 8:**
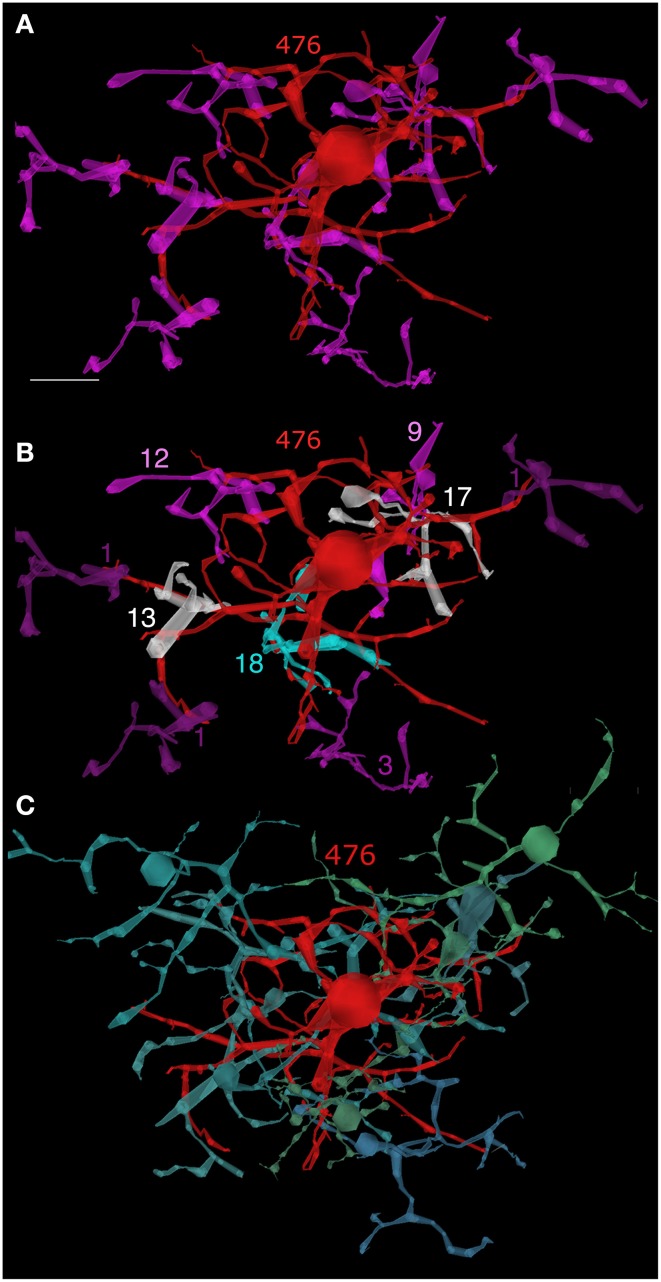
**AII cell 476—bipolar cell clusters. (A)** AII cell 476 (red) contacting nine rod bipolar cells (magenta). **(B)** Contact weights: 1 ribbon (dark purple), 3 ribbons (purple), 9–12 ribbons (magenta), 13–17 ribbons (white), 18 ribbons (cyan). **(C)** AII cell 476—ON cone bipolar cell clusters. Eight ON cone bipolar cells representing five of the eight known classes make multiple gap junctions with AII 476. Scale, 10 μm.

Mapping complete synaptic contacts is straightforward and the five AII cells contact between 9 and 17 rod bipolar cells (11.8 ± 3.3 SD, 0.28 coefficient of variation, CV). Their ribbon sampling is 7-fold more precise, however, averaging 75.6 ± 3 ribbons per AII cell with a CV of 0.04. Rod bipolar cells (Figure 7) are also precise in ribbon expression, with 31 ± 3.9 synaptic ribbons/bipolar cell (*n* = 27). The variation in rod bipolar cell contact seems to be completely geometric, representing the overlap of AII arboreal dendrites with rod bipolar cell axonal domains (Figures 8A,B). This difference in precision between cell sampling and synapse sampling is powerful. For a sixth AII cell to increase the synaptic CV to match the cell sampling CV, it would have to have a ribbon sampling rate 20 SDs larger. Conversely, for AII cell sampling to be as precise as synaptic sampling, the next 20 ACs counted would have to have SDs of zero. This argues that AII cells count synapses, not cells.

When AII amacrine cells encounter other cell classes, they make or decline connections by clearly stereotyped rules in their different compartments. The 39 AII amacrine cells in RC1 encounter each of 104 rod bipolar cells in multiple instances at multiple levels of the inner plexiform layer. Their arboreal dendrites are always postsynaptic to rod bipolar cells and never presynaptic, in 777 verified encounters from 1246 ribbons. And when lobules encounter rod bipolar cell axons in transit (in 6 validated instances) no ribbon contacts are formed, despite the fact that both rod bipolar cells do make axonal ribbons and will make synapses with AII arboreal dendrites high in the ON layer (Lauritzen et al., 2012). More importantly, AII cells can always distinguish between rod and ON cone bipolar cells by never making gap junctions with rod bipolar cells in the same encounters, and making gap junctions with every validated ON cone bipolar cell (*n* = 172 validated AII-CBb encounters). Similarly, AII lobular dendrites have their own rules. In contacts with 180 different OFF cone bipolar cells, they are presynaptic, postsynaptic, or both, but never form gap junctions, despite the fact that AII and CBa cells both express connexins. And while AII cell arboreal processes make extensive gap junctions with each other at every encounter (*n* = 525), rare instances of direct lobule-lobule contact (*n* = 6) do not show coupling, suggesting that AII connexins are excluded from lobules. Declined connections are more difficult to track, as they require first documenting a “touch” and then tracing both processes to ensure that a contact is not made elsewhere. By tracking the arboreal dendrites of AII cells in validated 11 touches with ganglion cells spanning many microns each, AII cells made no specializations (adherens, gap junctions, or synapses). In contrast, we have tracked an OFF α ganglion cell dendrite as it traversed the entire RC1 volume, encountering 23 separate AII lobules from 12 validated AII cells in its path. AII lobules also make abundant synapses onto verified GABAergic amacrine cells. Every lobule was presynaptic to the OFF α ganglion cell. But when lobules encounter AI GABAergic amacrine cell processes in the OFF layer (12 validated instances so far), they never make synapses, even though AI and AII amacrine cells make large adherens junctions at the arboreal level (Figure 5A). If we define AII contact errors as making gap junctions with rod or CBa bipolar cells, receiving ribbon from a CBb cell (with the exception of the CBb7 cell, described below), failing to accept ribbons from rod bipolar cells, or any variation of other detailed associations, we have documented 1773 proper connections (fully identifying both AII cells and the target cell) and 0 improper connections. We have also annotated and additional 3067 contacts between AII cells and targets not yet fully traced. In no case have we documented an obviously aberrant connection. These data suggest that AII cells are effectively errorless in executing their connectivity rules.

The interactions between AII cells and all other classes of neurons are too extensive to detail cell-by-cell, but can be tabulated (Table S1) and graphically summarized. There are 17 sign-conserving input partners to AII cells: nine glutamatergic and eight coupling. Volume RC1 contains at least six distinct classes of OFF cone bipolar cells and seven classes of ON cone bipolar cells (Marc et al., 2013, 2014) and all of them display partnerships with AII cells. All classes of OFF cone bipolar cells are both presynaptic and postsynaptic to AII cells, with postsynaptic events dominating by over 5-fold. Importantly, a single AII cell rejects input from most OFF bipolar cells and only a few ribbon inputs are permitted (see below).

**Figure 9:**
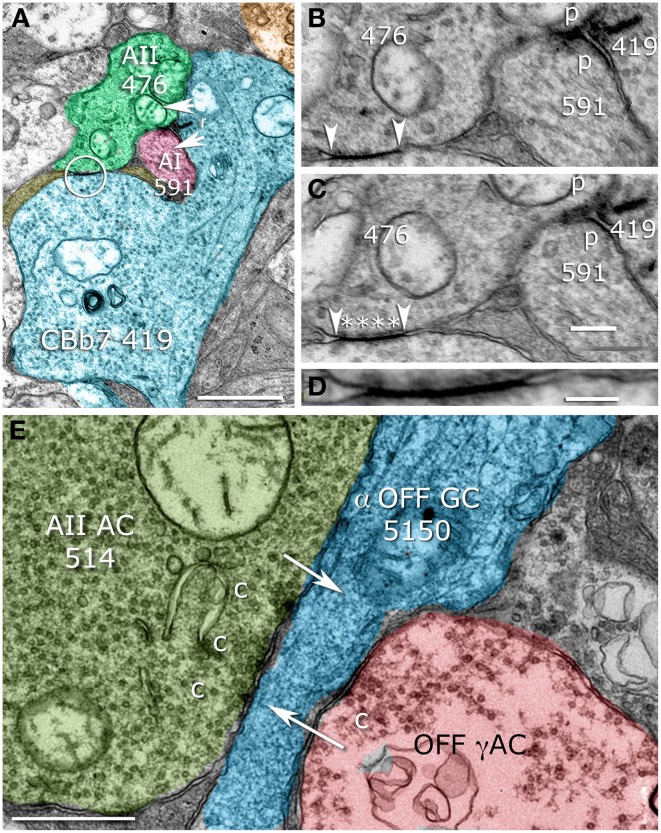
**Sparse AII cell contacts. (A)** CBb7 419 forming both ribbon synapses (r) and a gap junction (circle) with AII cell 476. CBb7 419 also contacts AI cell 591. **(B,C)** Serial sections through the ribbon synapse and its postsynaptic targets (p) and the gap junction (arrowheads) with characteristic AII cell cytoplasmic densities (Anderson et al., 2011b), asterisks. **(D)** Maximal resolution image of the gap junction showing apparent membrane fusion. **(E)** AII cell 514 making a multi-projection conventional presynaptic specialization (c) targeting OFF ganglion cell 5150 (arrow). Scales **(A,E)** 500 nm; **(C)** 250 nm, **(D)** 100 nm. **(B–D)** Were contrast adjusted to γ = 1.5 to discriminate gap junction membranes and cytoplasm.

All classes of ON cone bipolar cells are coupling partners with AII cells, but only in the waist or arboreal zone. Figure 8C displays the eight ON cone bipolar cell partners of AII 476, each of which forms multiple gap junctions with the AII at 28 sites: CBb3 6155 (1 gap junction), CBb3 4569 (9), CBb4w 170 (2), CBb4w 324 (1), CBb5w 483 (2), CBb4-5i 6156 (2), CBb5-6i 419 (8), CBb5-6i 4570 (3). While rod bipolar cells dominate the ON polarity input, wide-field, probable blue-sensitive (see Famiglietti, 2008) CBb7 bipolar cells are also presynaptic to AII cells and are unique in also being coupled via gap junctions (Figures 9A–D), which puts them in a position of being sparse amplifiers. We have partly analyzed seven CBb7 cells from a cohort of a dozen candidate cells that arborize close to the rod bipolar cells and are not part of the CBb3, 4, or 5 sheets. Every cell makes 1–3 three ribbon synapses and 2–7 gap junctions with neighboring AII cells (CBb 180 → AII As depolarizing rod signals invade CBb7 cells through gap junctions, they can immediately reinforce the signal with synaptic glutamate release. The significance of this small sample is nevertheless high: over 200 mapped non-CBb7 cone bipolar cells never make ribbon inputs AII cells despite making extensive numbers of gap junctions. Conversely all 7 CBb7 cells do make ribbons inputs (Komolgorov–Smirnov, α = 0.01, *p* = 5 × 10^−7^).

Finally, the dominant dopaminergic neuron of the mammalian retina is the TH1 (tyrosine hydroxylase positive type 1) axonal cell. It is also a glutamate neuron that is presynaptic to the neck region of AII amacrine cells (Anderson et al., 2011b) through very large synapses. The TH1 inputs are significantly larger than the aggregate OFF cone BC inputs and may dominate the photopic ON response of AII cells. This collection of sign-conserving inputs makes the AII cell a formal network hub, but the key to its function lies in synaptic weighting.

Inhibitory input to AII cells spans the retina and includes GABAergic amacrine cells of both OFF and ON varieties, and glycinergic ON and possible glycinergic ON-OFF amacrine cells. At least two classes of GABAergic OFF cells target AII lobules. While it is possible that some of the GABAergic input is also from ON-OFF cells, we have identified several classes of GABAergic ON-OFF amacrine cells that touch but explicitly fail to make any synaptic partnerships with AII cells. So, on balance, it appears that the GABAergic drive largely comes from monophasic cone-driven ON or OFF ACs. We have not dissected the weighting analyses of these subgroups, which will take at least another year of annotation, but they outnumber OFF bipolar cell ribbon synapses by >5-fold and outweigh them in synaptic area by >10-fold. Arboreal dendrites are targeted by GABAergic ON cells driven by cone bipolar cells as well as GABAergic AI amacrine cells in a feedforward motif. There are also narrow-field glycinergic amacrine cell inputs to arboreal dendrites from cone-driven ON and possible ON-OFF amacrine cells. Collectively, the inhibitory drive of the arboreal dendrites represents a cone → rod path inhibitory crossover; part of a collection of networks enabling cone signals that may suppress rod signals in the mesopic transition (Marc et al., 2013).

The synaptic outputs of AII cells are completely restricted to the lobules and target all OFF cone bipolar cell classes; GABAergic and glycinergic OFF amacrine cells that are also presynaptic to AI amacrine cells at large inhibitory sites on the proximal AI dendrites (Anderson et al., 2011b); and the dendrites of selected classes of retinal ganglion cells: specifically OFF α and δ ganglion cells (e.g., Figure 9E). These ganglion cell dendrites are very sparse and not every lobule encounters one. In contrast, every lobule encountered by these specific ganglion cells makes a synapse.

**Figure 10:**
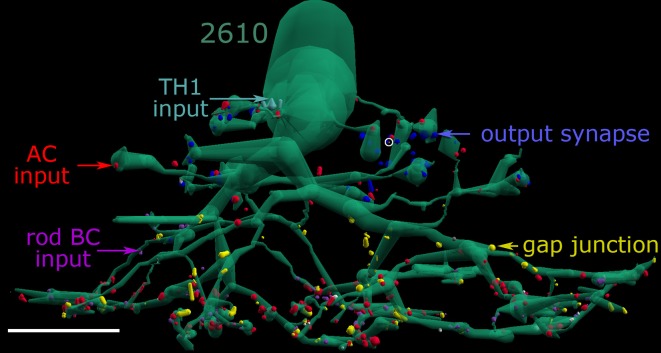
**AII cell partnerships**. AII cell 2610 with its rod bipolar cell input (magenta), amacrine cell input synaptic input (red), coupling sites (yellow), TH1 cell input (cyan), and glycinergic synaptic output (blue) dimensionally mapped onto its surface. Small white dots are adherens junctions. A single visible OFF cone BC ribbon is marked with a white circle. Scale, 10 μm.

**Figure 11:**
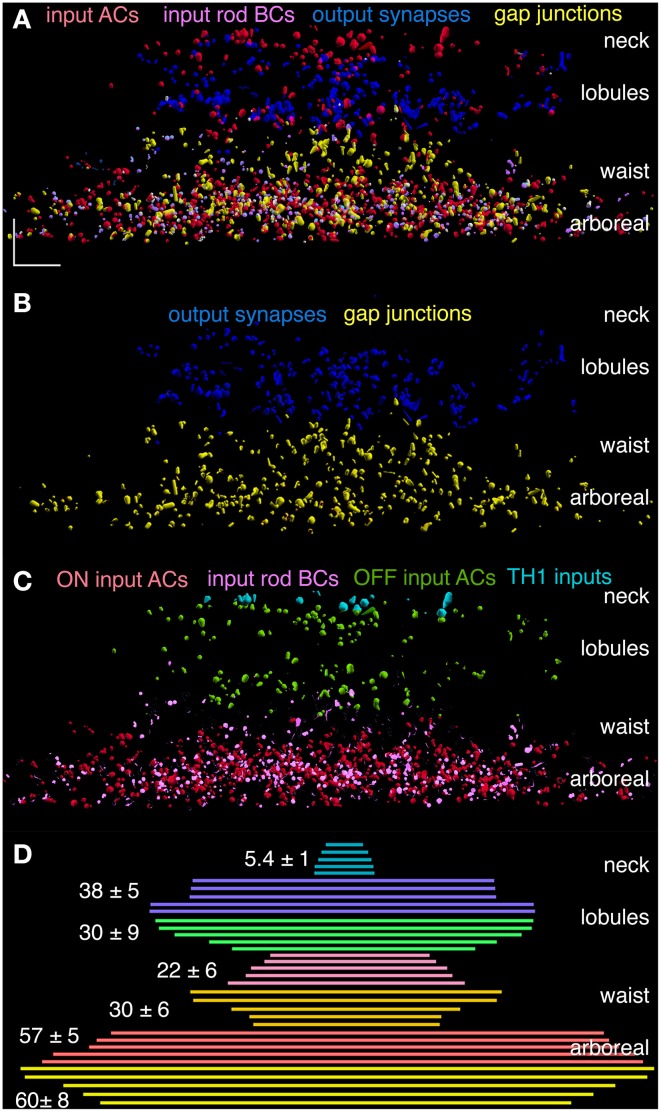
**Combined partner distributions for AII cells 410, 476, 514, 2610, and 3679. (A)** Superimposed AC synaptic input (red), rod BC ribbon input (magenta), coupling (yellow) and synaptic output (blue) mapped onto its surface with stratified anatomical features. **(B)** Outputs and coupling are completely segregated. **(C)** Inputs can be parsed into TH1 cell ON (cyan), OFF inhibition (green) and ON excitation (magenta) and ON inhibition (red). OFF excitation is so minimal that it is not visible in the plots. **(D)** The lateral extents of key stratified zones for each cell are centered and superimposed with the mean ± 1 SD width at left. The larger spread for the neck in C is caused by the misalignment of cell bodies with the center of the dendritic arbor. XY Scale, 10 μm.

Even with this diversity, we can develop a summarization of signal flow in the AII system. One approach involves mapping all the synapses associated with AII cells into five categories: rod bipolar cell input, off bipolar cell input, amacrine cell synaptic input, coupling and AII synaptic output. Figure 10 summarizes these partnerships for AII cell 2610 in dimensionally correct 3D positioning throughout the inner plexiform layer. Combining such partnership maps for all five cells generates a comprehensive view of the major signal flow architecture for the AII system. By aligning and superimposing all partnerships in the lateral XZ view (Z spans the IPL), the combined stratification profiles can be visualized (Figure 11A) and extracted into separate components on the same scale: outputs and coupling (Figure 11B), inputs (Figure 11C), and a summary of lateral spread (Figure 11D). The simultaneous visualization of outputs (blue) and coupling (yellow) demonstrates that the differential trafficking and functional assembly of presynaptic proteins for vesicle release into lobules and connexins for coupling into the waist and arboreal dendrites is errorless and defines the border between OFF (blue) and ON (yellow) layers of the inner plexiform layer, as first proposed by Kolb and Famiglietti (1974). And what we mean by errorless is that every CBb cell (>200 CBb cells making >1000 gap junctions) is always coupled to the AII cells it contacts, whereas no rod bipolar or CBa cell (>100 each) ever makes a gap junction. Similar counts can be had for all other classes of contacts. These inputs can be stratified into three simple zones (Figures 11B,C): excitatory ON inputs from TH1 cells (cyan), inhibitory OFF inputs (green), inhibitory ON inputs (red), and excitatory rod bipolar cell inputs (magenta). These can be further refined into finer classes but, for now, this analysis demonstrates how we can weight synaptic data from connectomics to build neuronal models. Weighting may require spatial rules as well, and by summarizing the lateral spread of each component we see that the interaction zones of the lobules and the waist are much narrower than the arboreal system. In fact, the lobular radius is only half the distance between cells, resulting in coverage of synaptic space without redundancy. Thus, the distal portion of the AII cell tiles retinal space and its lobules form a sampling grid with an approximate 10–15 μm spacing. The waist and neck subtile the space and their partnership patterns reflect the aggregation of wide-field TH1 cell signals, and the coupled ON cone bipolar cell network (Lauritzen et al., 2013). Finally the arboreal dendrites represent a center-to-center spatial covering, rather than a tile, with an ideal coverage factor of 4 (arboreal area/single cell area), which predicts that every AII cell should be 8-connected in a 2D grid. The measured homocellular coupling for eight AII cells whose arboreal dendrites have been completely mapped is 7.6 ± 0.9 partners.

**Figure 12:**
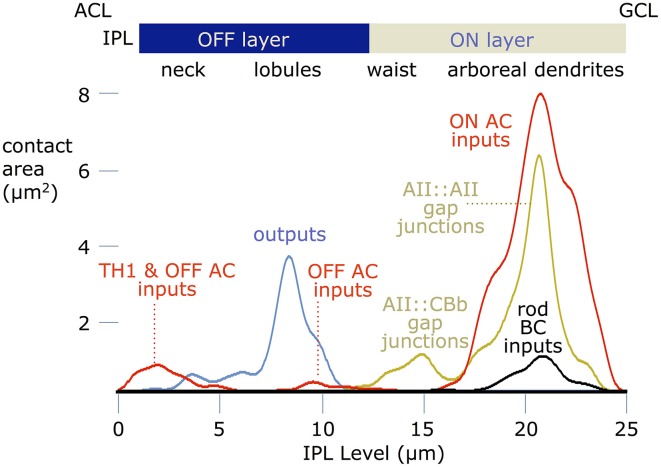
**Contact area profiles**. Contact areas were computed at every level of the inner plexiform layer (IPL), where 0 is the amacrine cell layer (ACL), and 25 is the ganglion cell layer (GCL). The ordinate scales presynaptic (outputs), postsynaptic (inputs) or gap junction contact areas for a typical AII cell. The abscissa is the depth of the inner plexiform layer. were computed for a lateral traverse through every level of the inner plexiform layer (IPL), where 0 is the amacrine cell layer and 25 is the ganglion cell layer.

These data can be summarized as synaptic area weightings (Figure 12). Partnership patterns were converted to binary images, capturing the location, number, and sizes of contacts, and profiled across the inner plexiform layer by averaging with over the width of the image. This computes the area of an input, coupling or output site and provides the spatial weights for modeling such connections. Based on prior descriptions of AII cells, we were surprised at first to find that inhibitory synapses (mostly ON GABAergic input) dominate the drive, but this is consistent with patterns of signal flow bipolar cells as well (Marc and Liu, 2000). The area of ON amacrine cell input is ≈8-fold higher that rod bipolar cell ribbon input. The integrated AII::AII coupling is approximately 6–7 μm^2^ while AII-CBb coupling represents ≈1 μm^2^. The lobular domain is dominated by output synapse areas, with minor OFF amacrine cell input and negligible OFF bipolar cell input. Finally the neck region is an approximate 1:1 mix of OFF amacrine cell and TH1 presumed ON synapses. Thus, the TH1 ON excitation is ≈1/3 the ON coupling excitation area.

**Figure 13:**
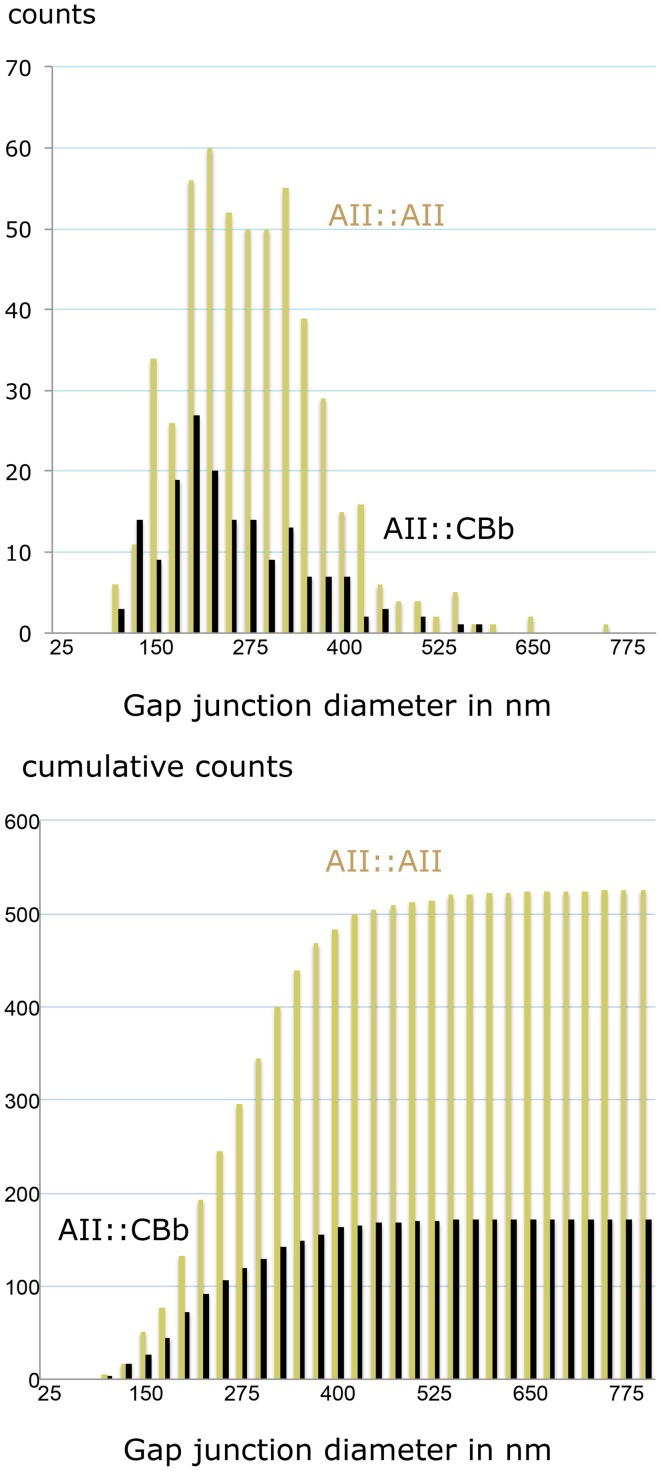
**Gap junction partnerships with AII cells. Top**, size distribution. **Bottom**, cumulative size distribution. Both histograms are binned in 25 nm increments. The cumulative frequency histograms are significantly different (Komolgorov–Smirnov, *p* = 0.0002).

We can also parse the strengths of individual gap junctions for homocellular AII::AII and heterocellular AII::CBb coupling. The RC1 database includes includes 525 AII::AII and 172 AII::CBb coupling instances (Figure 13), with a mean ± 1 SD gap junction diameter of 267 ± 95 nm for AII::AII and 238 ± 95 nm for AII::CBb pairings. The mean for heterocellular coupling is only about 11% smaller, but due to the large sample size is still highly significant (2 tailed homoscedastic *t*-test, *p* = 0.00052). More to the point, however, the largest gap junctions made by AII::AII pairings (745 nm) are 20% larger than made in AII::CBb pairings (592 nm) and the cumulative frequency distributions are significantly different. This corresponds to a 60% larger area for the largest homocellular versus heterocellular AII cell gap junctions.

## Discussion

### The AII cell is a dense hub

The partnerships of AII cells (Table S1) establish it as a network hub of high density and complexity. The AII cell contacts *every* retinal bipolar cell, which requires recognizing 15 cell classes with four differential contact rules: (1) exclusively postsynaptic for rod bipolar cells; (2) exclusively coupling for most CBb ON cone bipolar cells; (3) mixed coupling and postsynaptic for CBb7 bipolar cells; and both presynaptic and postsynaptic for all CBa OFF bipolar cells, albeit sparsely. Within the arboreal dendrite zone, AII cells can distinguish between rod and cone ON bipolar cells without error, being postsynaptic for the former and coupled to the latter. Further, the putative blue selective CBb7 makes both synapses and gap junctions, supporting the notion that unique bipolar cell surface markers facilitate synapse formation and that contact type and weight is a deterministic, not a stochastic process. This specificity, precise weighting, and consistent topology across AII is the antithesis of adjustable functional weighting of networks to compensate for variable connectivity (Prinz et al., 2004).

In detail, our conclusions regarding the numbers and variances of rod bipolar cells contacting a single AII cell are different from measurements of Tsukamoto and Omi (2013) in mouse, where their coefficients of variation (CV) are very similar for either number of rod bipolar cells or ribbon synapses contacting AII cells. This is almost certainly due to the small span of both mouse rod bipolar cell terminals and AII cells in the mouse (≈10 μm), while rabbit rod bipolar cell terminals span 20–30 μm and AII cells span 70 μm or more, meaning that a single rod bipolar cell is unlikely to dominate connectivity. Thus, it is quite unlikely that randomly placed AII cell in rabbit will have low CV. This provides a robust test for synaptic precision, which in rabbit is revealed to be high (very low CV).

Further, All afferent signal flow from photoreceptors is shaped by the AII transfer function. The 28 cell classes make 36 kinds of contacts, which is greater than the contact diversity reported for any other cell type in any nervous system. It also understates the complexity of AII cells, as many other classes touch AII cells, but functional contact is rejected. Given that there are ≈60 classes of neurons in the mammalian retina (Masland, 2001; Rockhill et al., 2002), the network graph of the retina shows that all neurons are within two hops from an AII cell, and almost half are directly connected. The scope of this connectivity, in turn, makes the entire retina a small-world system (Barthelemy and Amaral, 1999), despite its obvious dependence on multiple, tuned output channels manifest as ganglion cell diversity (Marc and Jones, 2002; Rockhill et al., 2002).

Further, the contact selectivity is completely regional in the AII cell. ON cone bipolar cell axons that touch AII lobules in the OFF layer never appear to form gap junctions, while all such axons that contact AII arboreal dendrites on the ON layer do so. This prevails despite the fact the ON cone bipolar cells can make functional presynaptic and postsynaptic specializations in the OFF layer (Dumitrescu et al., 2009; Hoshi et al., 2009; Lauritzen et al., 2012). It is also certain that we have underestimated the diversity of wide-field GABAergic amacrine cell interactions.

### The new AII network

**Figure 14:**
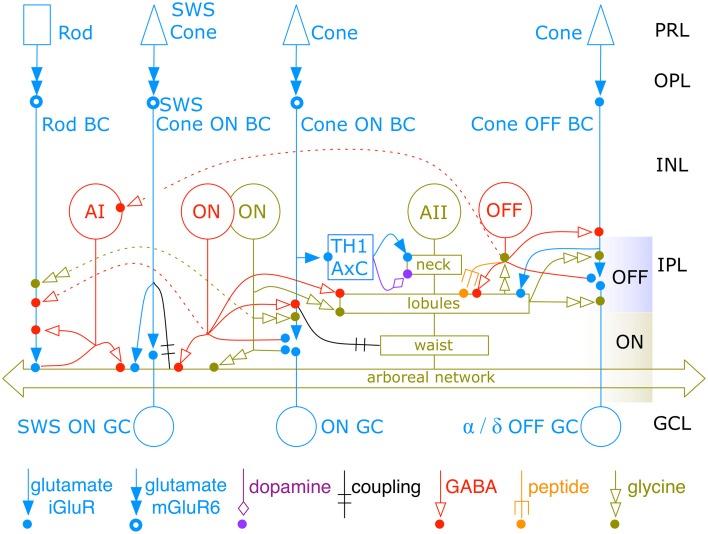
**The new AII amacrine cell network**. The complete AII cell network spans 4 classes of excitatory glutamate inputs, three coupling partners, dopamine modulation, three wide-field GABAergic inputs, peptide modulation, narrow-field glycinergic input, and outputs to OFF bipolar cells, OFF inhibitory neurons and two classes of OFF ganglion cells. All classes of CBb (except CBb7) and CBa bipolar cells are lumped into single ON and OFF channels. Multiple classes of ON and OFF amacrine cells are lumped into single representative classes. Dashed lines show paths for cone → rod suppressive crossover. TH1 AxC is a dual glutamate/dopamine wide-field axonal cell. PRL, photoreceptor layer; OPL, outer plexiform layer; INL, inner nuclear layer; IPL, inner plexiform layer; GCL, ganglion cell layer. Icon key at bottom denotes connection types. The OFF band captures the AII neck and lobules, which do not overlap with neighboring AII cells. The ON band captures the waist and the heavily coupled arboreal network, which overlaps other AII cells with a coverage factor of 4. Colored dots denote postsynaptic sites for each modality.

With this tabulation we can revise Figure 2 to form a richer network description of AII cells (Figure 14). There are six separate type of sign-conserving inputs to AII cells. Starting with the neck region, TH1 axonal cells make sparse conventional synapses on AII cells. While these cells were first thought to be dual GABAergic/dopaminergic neurons, small molecule profiling in the rabbit retina establishes that they have the same signature as glutamatergic ganglion cells and definitely lack any inhibitory signature (Anderson et al., 2011b). TH1 axonal cells are predominantly ON cells (Zhang et al., 2007) and receive direct ribbon input from en passant ON cone bipolar cell axons (Dumitrescu et al., 2009; Hoshi et al., 2009; Lauritzen et al., 2012), and their effect on AII cells should be an ON transient signal. The path from cone → ON BC → TH1 AxC → AII cell is purely glutamatergic, and given that the nominal gain of each transfer is some value *n* » 1 (Marc et al., 2013), this synaptic chain has an amplification proportional to *n*^3^ and may be the most sensitive photopic drive for AII cells. The low synapse number does not reduce this weighting significantly as the synaptic area is large. The second class of sign-conserving input is direct synaptic input from OFF cone bipolar cells (CBa cells) onto lobules. Despite the fact that every class of CBa cells makes some synapses onto AII lobules across the population, input to individual AII cells is low (2–5 synapses/cell) and the OFF ribbon synapse area weighting is approximately 20-fold lower than the third class of sign-conserving input, synaptic ribbons from rod bipolar cells. The fourth sign-conserving input is synaptic ribbon drive from CBb7 cells. We have begun mapping these cells and there are only seven validated cells so far in the entire RC1 volume (compared to 104 rod bipolar cells and over 200 CBb cells), as their arbors span well over 120 μm each. Nevertheless, their connectivities are unique and every one provides 1–3 ribbon inputs to a nearby AII cells. While this drive may be relatively weak compared to other inputs, it could dominate given the right stimulus conditions. The final two classes of sign-conserving input are heterocellular AII::CBb coupling and homocellular AII::AII coupling. Our profiling of gap junctions suggest that homocellular coupling is ≈7-fold stronger based on area than heterocellular coupling, assuming similar unitary connexin conductances. However, it appears that AII amacrine cell homocellular coupling is down-regulated by dopamine release and/or light adaptation (Mills and Massey, 1995; Bloomfield et al., 1997; Bloomfield, 2001) while heterocellular coupling is less strongly modulated. However, the regulation of AII::AII coupling may not be as simple as these early studies suggested (Hartveit and Veruki, 2012). In any case, AII::AII coupling may be attenuated in light adapted retinas, and the strength of AII::CBb coupling may become dominant. The notion that these gap junctions are differentially regulated is consistent with the fact that AII::AII coupling is completely dependent on connexin 36 (Cx36) expression, whereas AII::CBb coupling appears not to require Cx36 (Meyer et al., 2014).

AII::CBb coupling engages all classes of CBb ON cone bipolar cells. Thus, the physiological features of different types of CBb cells (e.g., Saszik and Devries, 2012) must readily be shared across the CBb::AII::CBb chain. How this plays out in ganglion cell drive based on targeting different bipolar cells remains to be clarified. Nevertheless, this supports a mechanistic emergence of selective connectivity patterns across cell classes, rather than a stochastic encounter-based connectivity, although whether this happens as a result of selective pruning or first intention or both remains to be resolved (Tian, 2011).

There are at least five major classes of AII interaction with inhibitory neurons. At the level of the lobules, AII cells are presynaptic and postsynaptic to two distinct classes of GABAergic feedback amacrine cells, one of which is peptidergic cell of unknown class that appears to make both conventional small vesicle and large peptide granule fusion sites on the lobules (Anderson et al., 2011b). AII lobule synaptic drive from amacrine cells is more prevalent than bipolar cell input by about 5-fold. Arboreal dndrites receive inhibition from at least three cells classes: sparse inputs from the classic AI amacrine cell and much more extensive inhibition from both wide-field cone-driven GABAergic and narrow-field glycinergic ON amacrine cells. Ultimately, each of these area weightings need to be combined with corresponding weights derived from physiological measures. The recent findings of Arman and Sampath (2012) suggest that this will be far from simple, but this is nevertheless the essential step in building a compact AII model.

We were not able to identify a unique axon-initial-segment process emerging from the AII neck as described by Cembrowski et al. (2012) using optical imaging or Tsukamoto and Omi (2013) using ultrastructure in the mouse. In rabbit AII cells, all lobular processes are long (15–20 μm), longer than the axon-initial-segment described in mouse. Every AII lobular processes displayed either large or small lobule like domains with vesicles, and both presynaptic and postsynaptic specializations. Often, one appendage was higher than most, emerging from the top of the neck, but we found no ultrastructural specialization that could be attributed to enhanced voltage-gated sodium channel expression. We noted that AII cells often display membrane densities similar to those described by Tsukamoto and Omi (2013), but have not observed that they are restricted to any compartment.

### Crossover

Heterocellular coupling to ON cone bipolar cells and glycinergic output to OFF cone bipolar cells appears to be a prime mechanism for redistributing amplified rod signals into cone pathways. The threshold of OFF ganglion cells in particular appears to be set by glycine release from AII cells (Muller et al., 1988; Arman and Sampath, 2012). But these pathways (and probably others) clearly operate at photopic levels as well (Manookin et al., 2008; Münch et al., 2009). Though the ability of ON cone bipolar cells to drive lobule output from arboreal dendrite input seems probable, Arman and Sampath (2012) provide evidence in mouse against the AII → OFF BC path being a dominant control arm in the scotopic state. This is at odds with our anatomic weighting. Perhaps the scenario is different in mouse, but in rabbit, every OFF BC receives significant glycinergic input from both AII cells and other glycinergic ACs. But in mouse, OFF BC light responses show no effect of glycine blockade. OFF ganglion cells receive many fewer glycine inputs than bipolar cells, but in mouse the influence of strychnine on threshold is more potent. The explanation for this is unclear. Either glycinergic ON to OFF crossover inhibition through bipolar cells (AII → OFF BC → OFF and ON-OFF ganglion cells) or direct mechanisms (AII → OFF α ganglion cell) could be operative at the photopic level (Molnar et al., 2009; Werblin, 2010). But the addition of glycinergic drive targeting inhibitory amacrine cells (Figure 14) that converge on the proximal dendrites of AI amacrine cells exposes an additional role for the AII cell in cone → rod crossover suppression networks (Marc et al., 2013). This may be a mechanism for fast mesopic switching between rod and cone vision and that operates by suppressing rod bipolar cell output when cone signals are dominant. The AII is also a recipient of cone-driven inhibition at the arboreal dendrite level (Figure 14), which may further suppress rod output signaling.

### Evolution

A distinguishing feature of mammals is the prevalence of rods (in most species) and the presence of the AII amacrine cell as a device to capture and amplify rod signals by driving them through a third ribbon synapse in the two arms of the cone pathway. As far as we know, non-mammalians do not exploit this mechanism and no homolog of the AII cell has yet been found, despite the abundance of narrow-field, multistratified and likely glycinergic amacrine cells in ectotherms. Most non-mammalians use mixed rod-cone bipolar cells as a merged scotopic-photopic mechanism to drive retinal ganglion cells (Ishida et al., 1980). Volume RC1 does provide some clues to the provenance of AII cells. If one removes all the rod components from the AII connectome (Figure 14), the vast majority of connections remain as an ON-OFF crossover system. Further, many non-mammalians (e.g., amphibians) display glycine signals in their ON bipolar cells (Marc et al., 1995; Yang and Yazulla, 1988), suggesting the presence of gap junctions between glycinergic amacrine cells and cone bipolar cells. Thus, the emergence of rod and rod bipolar cell proliferation in mammals could be the singular event that captured this crossover system as a scotopic amplifier (Dyer et al., 2009).

### Conflict of interest statement

Robert E. Marc is a principal of Signature Immunologics, Inc., manufacturer of some of the antibodies used in this work. The authors declare that the research was conducted in the absence of any commercial or financial relationships that could be construed as a potential conflict of interest.

## Supplementary material

Table S1 **A comprehensive list of all AII amacrine cell partnerships. sourceMarc-Frontiers-2014-Table-1.pdf**.
