# Supplementary material for: The AII amacrine cell connectome: a dense network hub
Source: Front Neural Circuits. 2014 Sep 4;8:104. doi: 10.3389/fncir.2014.00104 (PMC4154443; doi:10.3389/fncir.2014.00104)
Supplement: Table S1 — A comprehensive list of all AII amacrine cell partnerships. sourceMarc-Frontiers-2014-Table-1.pdf. [file Table1.PDF]

Table S1

AII AMACRINE CELL PARTNERS

Class	Input partners	Signaling
1	TH1 axonal cell	glutamate
2	CBa1w	glutamate
3	CBa1	glutamate
4	CBa1-2	glutamate
5	CBa2	glutamate
6	CBa2w	glutamate
7	CBab	glutamate
8	OFF γAC (feedback)	GABA
9	OFF pAC	GABA
10	OFF pAC	unknown peptide
11	ON GAC	glycine
12	ON-OFF GAC	glycine
13	ON γAC	GABA
14	AI AC	GABA
15	CBb7	glutamate
16	Rod BC	glutamate
	**Coupling partners**	
1	AII	
2	CBb3	
3	CBb4w	
4	CBb3-4i	
5	CBb4-5i	
6	CBb5-6i	
7	CBb5w	
8	CBb6	
9	CBb7	
	**Synaptic targets**	
1	CBa1w	glycine
2	CBa1	glycine
3	CBa1-2	glycine
4	CBa2	glycine
5	CBa2w	glycine
6	CBab	glycine
8	OFF γAC motif C2	glycine
9	OFF GAC motif C2	glycine
10	α OFF GC	glycine
11	δ OFF GC	glycine
